# Efficacy of Inhaled N-Chlorotaurine in a Mouse Model of *Lichtheimia corymbifera* and *Aspergillus fumigatus* Pneumonia

**DOI:** 10.3390/jof8050535

**Published:** 2022-05-20

**Authors:** Cornelia Speth, Günter Rambach, Andrea Windisch, Magdalena Neurauter, Hans Maier, Markus Nagl

**Affiliations:** 1 Institute of Hygiene and Medical Microbiology, Medical University of Innsbruck, A-6020 Innsbruck, Austria; cornelia.speth@i-med.ac.at (C.S.); guenter.rambach@i-med.ac.at (G.R.); andrea.windisch@i-med.ac.at (A.W.); magdalena.neurauter@i-med.ac.at (M.N.); 2 Christian-Doppler Laboratory for Invasive Fungal Infections, Medical University of Innsbruck, Schöpfstraße 41, A-6020 Innsbruck, Austria; 3 INNPATH GmbH-Institute of Pathology, A-6020 Innsbruck, Austria; hans.maier@innpath.at

**Keywords:** N-chlorotaurine, mouse, pneumonia, molds, *Lichtheimia*, *Aspergillus*, inhalation, respiratory system, lung, antiseptic, anti-infective

## Abstract

N-chlorotaurine (NCT) can be used topically as a well-tolerated anti-infective at different body sites. The aim of this study was to investigate the efficacy of inhaled NCT in a mouse model of fungal pneumonia. Specific pathogen-free female C57BL/6JRj seven-week-old mice were immune-suppressed with cyclophosphamide. After 4 days, the mice were inoculated intranasally with 1.5 × 10E7 spores of *Lichtheimia corymbifera* or 1.0 × 10E7 spores of *Aspergillus fumigatus*. They were randomized and treated three times daily for 10 min with aerosolized 1% NCT or 0.9% sodium chloride starting 1 h after the inoculation. The mice were observed for survival for two weeks, and fungal load, blood inflammation parameters, bronchoalveolar lavage, and histology of organs were evaluated upon their death or at the end of this period. Inhalations were well-tolerated. After challenge with *L. corymbifera*, seven out of the nine mice (77.8%) survived for 15 days in the test group, which was in strong contrast to one out of the nine mice (11.1%) in the control group (*p* = 0.0049). The count of colony-forming units in the homogenized lung tissues came to 1.60 (1.30; 1.99; median, quartiles) log_10_ in the test group and to 4.26 (2.17; 4.53) log_10_ in the control group (*p* = 0.0032). Body weight and temperature, white blood count, and haptoglobin significantly improved with NCT treatment. With *A. fumigatus*, all the mice except for one in the test group died within 4 days without a significant difference from the control group. Inhaled NCT applied early demonstrated a highly significant curative effect in *L. corymbifera* pneumonia, while this could not be shown in *A. fumigatus* pneumonia, probably due to a too high inoculum. Nevertheless, this study for the first time disclosed efficacy of NCT in pneumonia in vivo.

## 1. Introduction

N-chlorotaurine (NCT), the N-chloro derivative of amino acid taurine, is a long-lived oxidant produced by activated human leukocytes during an oxidative burst [1,2]. Naturally, it is formed via a reaction of a strong oxidant hypochlorous acid (HOCl) with taurine [1,2]. This leads to detoxification of the highly reactive HOCl, which is transferred to the low-reactive and mild oxidant NCT [3,4]. Further functions of NCT are antimicrobial activity and contribution to termination of inflammation due to its anti-inflammatory properties, such as downregulation of several cytokines and chemokines, nuclear factor kappa B, prostaglandins and upregulation of hemoxygenase-1 and Nrf-2 [5,6,7,8].

NCT can also be synthesized chemically as a crystalline sodium salt (molecular weight, 181.57 g/mol) [9], which has been established in pharmaceutical quality at our laboratory. Aqueous and buffer solutions can be prepared instantaneously and demonstrated broad-spectrum microbicidal activity against bacteria, viruses, fungi, and protozoa [10,11]. Further development of NCT as an antiseptic and an anti-infective was demonstrated in clinical phase II studies on topical application of a 1% aqueous solution of NCT in infections of the eye, the ear, the skin, the oral cavity, which showed efficacy throughout and in case series for several other indications such as nose and throat, herpes, and urinary tract infections [10,11,12,13].

In the recent years, tolerability and safety of inhalation of NCT was investigated in detail. No adverse effects by 1% NCT were seen in the healthy lung of piglets and mice and in the inflamed lung of piglets [14,15,16]. A phase I clinical study in humans with daily inhalations of 1% NCT for 5 days confirmed tolerability with only slight sensations, i.e., chlorine taste and occasional tickling in the throat, and no changes of lung function parameters such as forced expiratory volume in 1 s [17]. The next logical step in this development is to demonstrate the tolerability, safety, and efficacy of inhaled NCT in pneumonia. We have repeatedly used and established a mouse fungal pneumonia model at our facility. Thereby, mold spores are instilled into the nose of immune-suppressed mice, which leads to pneumonia and systemic infection within short time [18]. 

The aim of this study was to investigate the efficacy, tolerability, and safety of rapidly applied inhaled 1% NCT in the mouse mold pneumonia model.

## 2. Materials and Methods

### 2.1. Test and Control Solutions for Inhalation

The NCT sodium salt was manufactured in pharmaceutical quality [9], lot 27 April 2021, 99.7% potency, sterile and pyrogen-free. The 1% (55 mM) NCT test solution for inhalation was prepared in sterile and pyrogen-free distilled water (Fresenius Kabi GmbH, Friedberg, Germany, lot 19QAA900). As a control solution, 0.9% sodium chloride for injection was used, also from Fresenius Kabi GmbH, lot 19PKA300.

### 2.2. Other Chemicals

Supplemented minimal medium (SUP) agar contained 16 g Difco^TM^ Agar Granulated, 5 g yeast extract (Sigma-Aldrich, local branch, Vienna, Austria), 10 g glucose, 1 g NH_4_Cl, 0.25 g MgSO_4_ heptahydrate, 4 g KH_2_PO_4_, and 0.9 g K_2_HPO_4_ per liter (*w*/*v*).

### 2.3. Molds and Growth Media

*Lichtheimia corymbifera* (CBS 109940, internal number LC9) was a patient isolate derived from a clinical specimen and confirmed by sequencing the internal transcribed spacer (ITS) region of the ribosomal genes [19]. *Aspergillus fumigatus* (internal number A22) was a clinical isolate, too, obtained from a lung biopsy specimen of an immunosuppressed patient with respiratory insufficiency [20]. The fungi were deep-frozen for storage. *L. corymbifera* was grown on SUP agar, *A. fumigatus*—on Sabouraud agar (BD Diagnostic Systems) for 5 days at 37 °C each [18,21]. Conidia were harvested with PBS plus 0.05% Tween 20 from the plates, washed with 0.9% NaCl, and filtered through a 45 μm and a 10 μm cell strainer (BD Diagnostics System). After counting with a hemocytometer, the spore suspension was adjusted to 7.5 × 10^8^/mL spores of *Lichtheimia corymbifera* and 5.0 × 10^8^/mL spores of *Aspergillus fumigatus* in 0.9% NaCl with 0.05% Tween 20 and used immediately for intranasal infection of the animals [18]. Stock solutions were preserved at 4 °C or, for longer time periods, frozen at –80 °C. 

### 2.4. Inhalation Device

For inhalation, up to six mice were placed in a transparent plastic chamber of 23 cm × 20 cm × 10 cm (induction chamber, PS-0346, Rothacher Medical GmbH, Heitenried, Switzerland) connected with an evacuative tube. An afferent tube (PS-0582, Rothacher) was connected with a Pari Boy^®^ SX nebulizer (PARI GmbH, Starnberg, Germany), which produced aerosol particles with a median mass diameter of 2.9 μm (specification of the manufacturer) [15]. Immediately before inhalation, the mice were placed into the chamber. NCT or saline (3 mL each) were added to the reservoir of the nebulizer. After starting the nebulizer, the chamber was filled with the aerosol within a few seconds, and the single inhalations were performed for 10 min, consuming about 2 mL of the solution. Subsequently, the mice were placed back into their cages. 

### 2.5. Animal Ethics Statement

The Central Laboratory Animal Facility of the Medical University of Innsbruck and all the experimental procedures of the study complied with the Austrian Animal Experimental Act (BGBl. I No. 114/2012). All the animal experiments were approved by the National Committee for Animal Care of the Austrian Federal Ministry of Science, Research, and Economy (BMWFW) (approval No. 2021-0.338.269). Blood sampling and euthanasia of the mice were performed under isoflurane anesthesia, and all efforts were made to minimize suffering.

### 2.6. Mice, Test Groups, and Treatment

Specific pathogen-free female C57BL/6JRj seven-week-old mice (102 animals) were purchased from Janvier Labs (Saint Berthevin Cedex, France). The mice were kept in polyetherimide cages at 22 °C with laboratory bedding, nesting material, gnawing sticks (ABEDD, Vienna, Austria), and mouse shelters (EHRET, Tulln, Austria). In the housing room, the photoperiod was adjusted to 12 h, the air exchange rate—to 12 times per hour. The mice were fed with normal mouse chow (pellets, ssniff laboratory diets, Soest, Germany) and water ad libitum.

The defined humane endpoints included body weight loss of more than 20%, decrease in the average body surface temperature of more than 2.5 °C, disturbed or missing feeding/drinking, dyspnea (inspiratory/expiratory stridor, agonal respiration), and neurological symptoms such as abnormal body posture (body/head tilt), impaired mobility, walking in circles, or body rotation. Each mouse meeting an endpoint criterion was immediately anesthetized by isoflurane inhalation and euthanized by cervical dislocation.

The mice were randomized into eight groups after arrival (Table 1). All the animals were housed and observed for 1 week without treatment. On day minus 6, their body weight was measured. Immune suppression with intraperitoneal 100 mg/kg cyclophosphamide started on day minus 4 in groups 3–8 and was performed every third day until euthanasia, maximally until day 11. On day zero, the mice were infected intranasally under isoflurane anesthesia with aliquots of 20 µL PBS plus 0.05% Tween 20 containing 1.5 × 10^7^ spores of *Lichtheimia corymbifera* (groups 5–6) or 1.0 × 10^7^ spores of *Aspergillus fumigatus* (groups 7–8). The numbers of spores were chosen according to our previous experience with the model [18].

Inhalations started 1 h after inoculation of fungal spores in groups 5–8 and were performed 3 times daily for 10 min each from day zero to day 14 in all the groups with either 1% NCT (test groups) or 0.9% NaCl (control groups).

### 2.7. Evaluation Parameters

Body weight and body surface temperature were measured daily from day minus 4 to the end of the study in all the animals. Clinical evaluation (comprising reduced food/water uptake, apathy, rigidness, weight loss, and heavy breathing) was performed daily, too, over the whole period. Humane endpoints and survival were monitored daily.

From five mice of groups 3–8, on day 2, blood was taken from the submandibular vein (100–150 µL) using EDTA as an anticoagulant. 

In three of these animals, after sacrifice, the lungs, the brain, the spleen, and the kidneys were dissected. For determination of the fungal load, the organs were frozen in liquid nitrogen and stored at –20 °C. For histology, the organs were fixed in 4% formalin. 

In two of these animals, bronchoalveolar lavage (BAL) was performed on day 3 subsequent to euthanasia in isoflurane anesthesia.

From blood, hemograms were determined automatically using a blood cell counter (animal blood counter Vet abc, Scil). All the parameters of a complete blood cell count were obtained: white blood cells (WBC) and differential WBC (neutrophils, eosinophils, basophils, lymphocytes, and monocytes), erythrocytes, hemoglobin, hematocrit, platelets, mean corpuscular volume, mean corpuscular hemoglobin, and mean corpuscular hemoglobin concentration. Haptoglobin as a meaningful inflammation parameter was determined using a commercial ELISA according to the instructions of the manufacturer (R&D Systems, Inc., Minneapolis, MN, USA) [22].

BAL in euthanized mice was performed as previously published [23]. The trachea was exposed by removing the salivary glands and the outer muscle layer. A 24G catheter (Braun, Melsungen, Germany) was inserted between two cartilage rings. A syringe was used to inject 0.7 mL of a buffer solution (Hanks Balanced Salt Solution; Life Technologies, Paisley, UK) into the lung. After gently massaging the thorax of the mouse, the solution was aspirated and collected. This procedure was repeated twice more. The samples were centrifuged at 400× *g* for 7 min at 4 °C. The supernatant was discharged and the erythrocytes were lysed in an ammonium–chloride–potassium (ACK) red blood cell lysis buffer [23]. After washing in PBS, the cells were fixed with methanol subsequent to cytospin centrifugation for 7 min at 108× *g*. Staining was performed with May-Grünwald and Giemsa.

For histology, sections of the organs were subsequently stained with a combined hematoxylin and eosin/methenamine silver stain [24].

### 2.8. Determination of the Fungal Load with an Improved New Homogenization Method

Since previous methods for organ homogenization with a rotating knife on a stirring stick or by mechanical treatment followed by MixerMill were laborious and consumed much time, we first evaluated and then established a much more rapid and easier method [18,21]. A single whole organ deep-frozen and transported in liquid nitrogen to the laboratory was placed into a Lysing Matrix M 2 mL tube containing one ceramic ball with 6.4 mm diameter (MP Biomedicals Germany GmbH, Eschwege, Germany), and 1 mL of PBS was added. The organ was thawed in the tube in an incubator at 37 °C for a short time (about 5 min) before the tube was placed on ice for short-term storage. Subsequently, the samples in these tubes were homogenized in a FastPrep-24 5G homogenizer (MP Biomedicals Germany GmbH) at 4 m/s for 20 s. Pilot tests with different homogenization times showed that these conditions were sufficient for complete homogenization of the lungs, brain, spleen, and kidneys of the mice. Subsequently, the samples were placed on ice again and vortexed three times for 3 s. Aliquots of 400 µL were removed carefully to avoid the transfer of organ clumps and diluted in 400 µL 0.9% NaCl. This step turned out to be of advantage to avoid blockage of the small tubes of the spiral plater. Quantitative cultures were performed routinely from these dilutions. Aliquots of this suspension were spread onto Sabouraud agar for *A. fumigatus* or SUP agar plates for *L. corymbifera* in duplicate (50 µL each) using an automatic spiral plater (model WASP 2; Don Whitley, Shipley, UK). The detection limit from the homogenate was 20 CFU/mL, taking into account both plates and the previous twofold dilution in 0.9% NaCl. The plates were grown for 24 h at 37 °C for *A. fumigatus* and 20 °C (to avoid overgrowth) for *L. corymbifera* before the colonies were counted. The plates with absence of fungal colonies were grown for 3 days before definite evaluation. 

A previous pilot test demonstrated the survival of the homogenization procedure by both *A. fumigatus* and *L. corymbifera* in the hyphal form. Spores of both species (2 × 10^4^/mL) were incubated separately in a Sabouraud broth overnight at 37 °C. Separate samples (750 µL each) were homogenized in Lysing Matrix M 2 mL tubes as mentioned above at 4 m/s in a FastPrep-24 5G homogenizer for 5, 10, 15, 20, 25, and 30 s, while two samples for each species were not homogenized. Aliquots of 400 µL of the samples were diluted with 400 µL 0.9% saline. After performing quantitative cultures with the spiral plater and incubation mentioned above, CFU counts were identical in the homogenized and non-homogenized samples.

For *A. fumigatus*, the control counts were 2.89 and 3.12 log_10_ CFU/mL and the homogenized ones ranged between 3.31 and 3.68 log_10_ CFU/mL, with no differences between the single homogenization times. For *L. corymbifera*, the control counts were 4.65 log_10_ CFU/mL in both controls and the homogenized counts ranged between 4.48 and 4.79 log_10_ CFU/mL. In another pilot validation test, mouse kidneys were spiked separately with spores of each species, stored at 37 °C overnight, cut into two identical pieces, of which one was homogenized with the new method for 20 s in a FastPrep-24 5G homogenizer, and the other one—with a stick homogenizer for 60 s needed for good homogenization. The count of quantitative cultures was 72 and 76 CFU after FastPrep and 51 and 56 CFU after the stick homogenizer. 

### 2.9. Statistics

The data are presented as the medians and quartiles because of non-Gaussian distribution. Mann–Whitney U test in case of two groups or Kruskal–Wallis test in case of more than two groups were used to test for statistical difference. Survival curves were compared using the logrank Mantel–Cox test. The frequencies were compared with Fisher’s exact test. A *p* value of < 0.05 was considered significant for all the tests. Calculations were performed with the GraphPad Prism 8.0.1 software (GraphPad Inc., La Jolla, CA, USA).

## 3. Results

### 3.1. Tolerability of NCT

The inhalations were tolerated well by all the animals in all the groups. There were no indications of immediate or delayed discomfort by NCT compared to saline. No differences in all the parameters could be found between groups 1 and 2, which were neither treated with cyclophosphamide nor with fungi but only with inhalations of saline and NCT, respectively. All these mice survived for 14 days without detectable discomfort.

### 3.2. Efficacy of NCT

**Figure 1:**
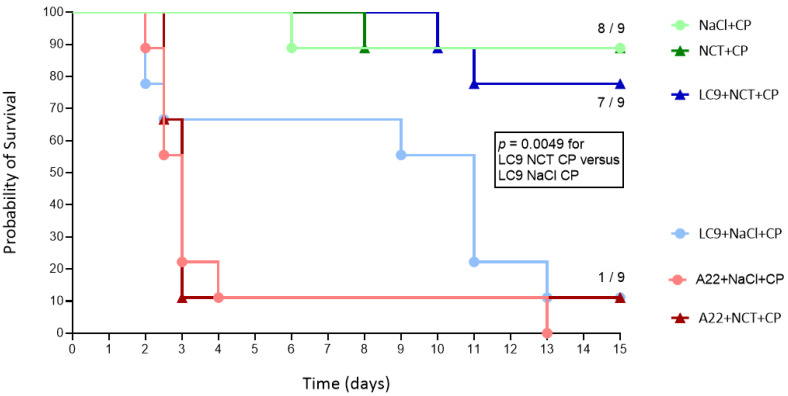
Survival curves of the immunosuppressed mice after mold lung infection by nasal inoculation with 1.0 × 10^7^ spores of *Aspergillus fumigatus* or 1.5 × 10^7^ spores of *Lichtheimia corymbifera* and treatment with inhaled 1% NCT or 0.9% NaCl (*n* = 9 each). The control mice that did not receive fungi but cyclophosphamide and inhalations with NCT or saline are also shown and survived except for one mouse in the saline group that lost weight massively and one mouse in the NCT group that accidentally died after blood-taking; *p* = 0.0049 between NCT and NaCl for *L. corymbifera*; *p* = 0.568 between NCT and NaCl for *A. fumigatus*; *p* = 0.587 between the controls without fungi and NCT with *L. corymbifera* (logrank Mantel–Cox test each). All the mice treated with inhalations of NCT or saline without cyclophosphamide (*n* = 9 each) survived (not shown). CP, cyclophosphamide; A22, *A. fumigatus*; LC9, *L. corymbifera*.

There was a marked difference between *L. corymbifera* and *A. fumigatus* (Figure 1). While in the mice challenged with *L. corymbifera*, a strong and highly significant effect on multiple parameters was found, in those challenged with *A. fumigatus*, no significant therapeutic effect could be detected. The most likely explanation in our opinion is that the number of spores in the inoculum was too high with *A. fumigatus* and led to an overwhelming rapid infection that could not be influenced, while with *L. corymbifera*, the inoculum led to a more decelerated infection ideal to demonstrate the effect of NCT.

### 3.3. Survival of the Mice

In the mice inoculated intranasally with 1.5 × 10^7^ spores of *L. corymbifera*, the infection was more delayed than with *A. fumigatus* so that the effect of NCT could be detected. In the *L. corymbifera* control group, eight of the nine mice died after 13 days and one survived. By contrast, in the NCT group, one mouse died on days 10 and 11 each, while seven survived for 15 days (*p* = 0.0049 between the test and control groups, Figure 1).

The mice challenged intranasally with 1.0 × 10^7^ spores of *A. fumigatus*, however, rapidly developed a severe pulmonary infection, and eight of the nine mice monitored for 15 days died after 2–4 days in both the test and control groups. The last mouse of the control group died after 13 days, while one of the nine of the test group survived (*p* = 0.568, Figure 1).

All the mice treated with cyclophosphamide and inhalations of NCT or NaCl, but not with fungi, survived except for one mouse in each group. The mouse in control group 3 became apathic, trembled, and lost 6 g of weight on day 6 so that it had to be euthanized due to intolerance to cyclophosphamide treatment. The mouse in NCT group 4 had no conspicuous parameters or behavior, but accidentally died after blood-taking on day 8. All the nine mice treated with plain inhalations of NCT or NaCl (groups 1–2) were monitored for 14 days and survived without detectable discomfort.

### 3.4. Fungal Load in Organs

The number of CFU counts in homogenized organs was determined in 12 mice each of groups 5–8, whereby three mice in each group were euthanized two days after inoculation, while the others were investigated upon death or after 15 days. The main fungal load could be detected in the lungs for both fungal species, while only low numbers or zero were found in other organs. 

**Figure 2:**
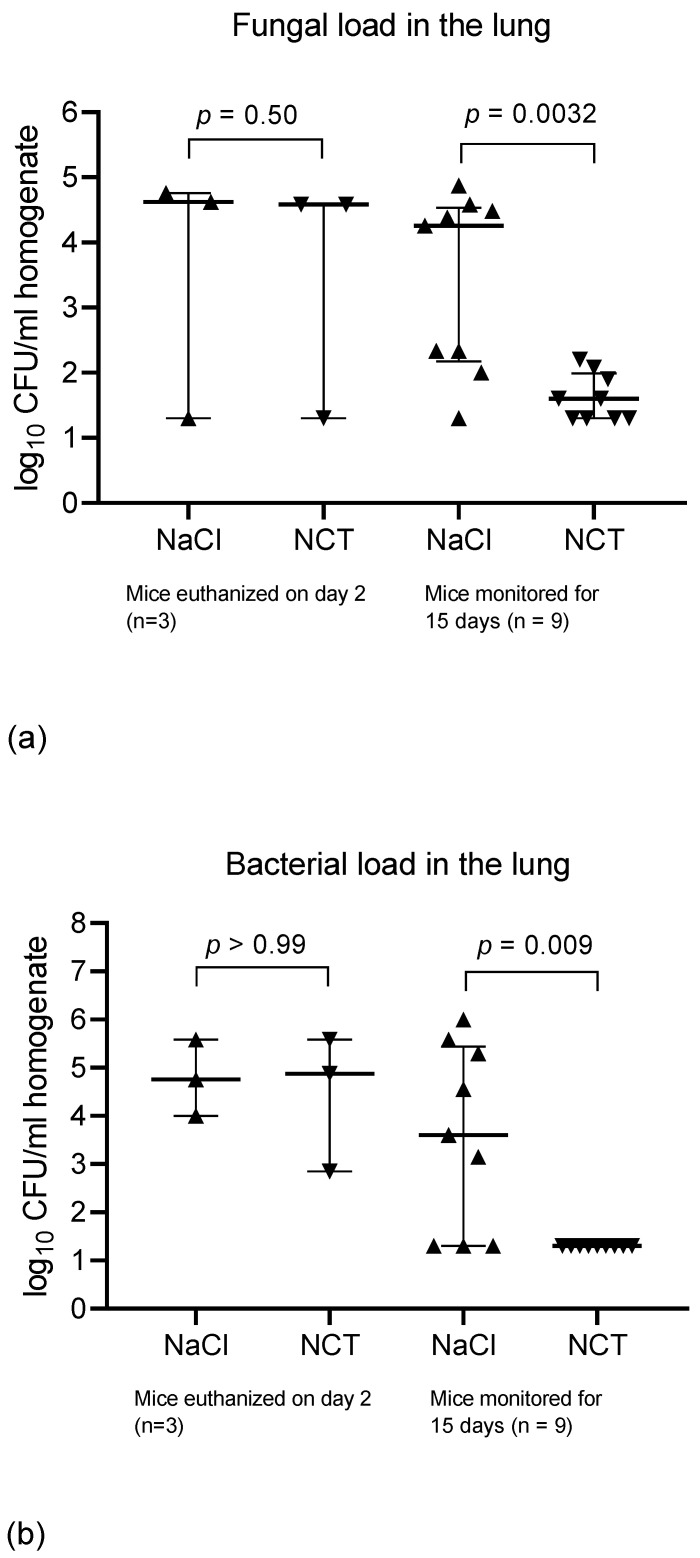
Pathogen counts in the lung of the mice challenged with *L. corymbifera*. Fungal (**a**) and bacterial (**b**) counts in the homogenized lung of the mice euthanized on day 2 and of the mice monitored for survival for 15 days. Scatter dot plots with medians and quartiles, Mann–Whitney test.

In the mice infected with *L. corymbifera*, the fungal load in the lungs was 4.32 (2.09; 4.61; median, 25% and 75% percentiles) log_10_ CFU/mL of the homogenized organ (range, 1.30–4.87 log_10_ CFU/mL, *n* = 12) in the control group. In the test group, it was 1.60 (1.30; 2.17, median, 25% and 75% percentiles) log_10_ CFU/mL (range, 1.30–4.58 log_10_ CFU/mL). The difference between both groups was significant (*p* = 0.025). Of these, single mice euthanized on day 2 had 4.62, 4.76, and < 1.30 log_10_ CFU/mL fungi in their lungs in the control group, and 4.58, 4.58, and <1.30 log_10_ CFU/mL in the test group, with no difference between the groups (Figure 2a). Therefore, the overall difference in the fungal counts resulted from the nine animals of each group, which were not euthanized on day 2 but monitored for 15 days. The cultures from these mice revealed a fungal load of 4.26 (2.17; 4.53; median, 25% and 75% percentiles) log_10_ CFU/mL with a range of 1.30–4.87 log_10_ CFU/mL in the control group. In the test group, it was only 1.60 (1.30; 1.99; median, 25% and 75% percentiles) log_10_ CFU/mL with a range of 1.30–2.20 log_10_ CFU/mL. This difference was highly significant (*p* = 0.0032) (Figure 2a).

Regarding the other organs, only 1 CFU (1.30 log_10_ CFU/mL) of *L. corymbifera* grew in the spleen of one mouse in the test group. By contrast, in the control group, the fungus grew in three mice in the spleen at counts of 2.30, 3.86, and 2.78 log_10_ CFU/mL, in five mice in a kidney (1.30, 2.20, 2.85, 1.30, and 1.60 log_10_ CFU/mL), and in three mice in the brain (1.30, 1.78, and 1.60 log_10_ CFU/mL). The frequency of positive cultures (1/36 versus 11/36) was highly significant between both groups, with *p* = 0.003. 

Cultures for *L. corymbifera* from homogenized organs were performed with SUP agar, which does not suppress bacterial growth in contrast to Sabouraud agar, which was used for *A. fumigatus* and contained antibiotics chloramphenicol and gentamicin. Of note, we found additional bacterial growth in many cultures from organs of the mice treated with *Lichtheimia*, presumably because the mice were immunocompromised. The strains identified were *Klebsiella pneumoniae*, *Escherichia coli*, and, at lower counts, *Staphylococcus aureus*. Bacteria were found in 9 of the 12 mice in the lung in the control group at a range of 3.15–> 6.0 log_10_ CFU/mL (4.76; 3.80, 5.58; median, 25% and 75% percentiles), in 5 of the 12 mice in a kidney at a range of 2.15–4.20 log_10_ CFU/mL, in 3 of the 12 mice in the spleen with 2.58, 2.72, and 5.58 log_10_ CFU/mL, and in one mouse in the brain with 3.90 log_10_ CFU/mL. By contrast, in the test group, only the three mice euthanized on day 2 had bacteria in their lungs (2.85, 4.88, and 5.88 log_10_ CFU/mL), while all the other cultures remained negative for bacteria. The frequency of positive cultures in all the organs (3/48 versus 18/48) was highly significant between both groups, with *p* = 0.0004 (Fisher’s exact test). The bacterial load in the lungs had a very similar pattern to that of the fungal one, with a highly significant difference between the test and control groups in the mice monitored for survival over 15 days (Figure 2b).

In the mice infected with *A. fumigatus*, the fungal load in the lungs was 2.95 (2.36; 3.16; median, quartiles, *n* = 12) log_10_ CFU per mL of the homogenized organ in the control group. In the test group, it was 2.67 (2.47; 3.25; median, quartiles, *n* = 12) log_10_ CFU/mL. The values for both groups were similar (*p* = 0.989). The mice euthanized on day 2 (*n* = 3) had similar counts as the mice monitored for 15 days (*n* = 9). Single exceptions with low counts were the one mouse in the control group that died on day 13 with 1.30 log_10_, the one mouse in the test group euthanized on day 2 with 1.30 log_10_, and the one mouse in the test group that survived with no fungus detectable after 15 days. In one different control mouse each, 1.30 log_10_ in the brain, and 1.90 log_10_ in the kidney were detected. All other fungal cultures of the brain, kidney, and spleen remained free of *A. fumigatus* growth.

### 3.5. Organ Weights

**Figure 3:**
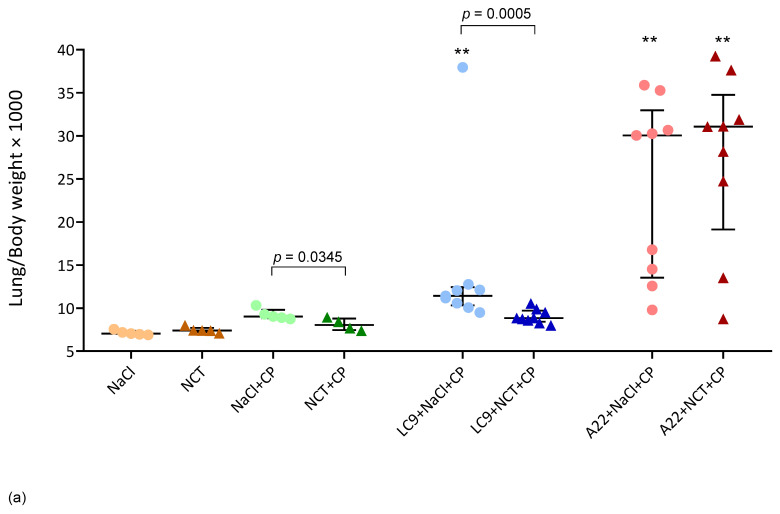

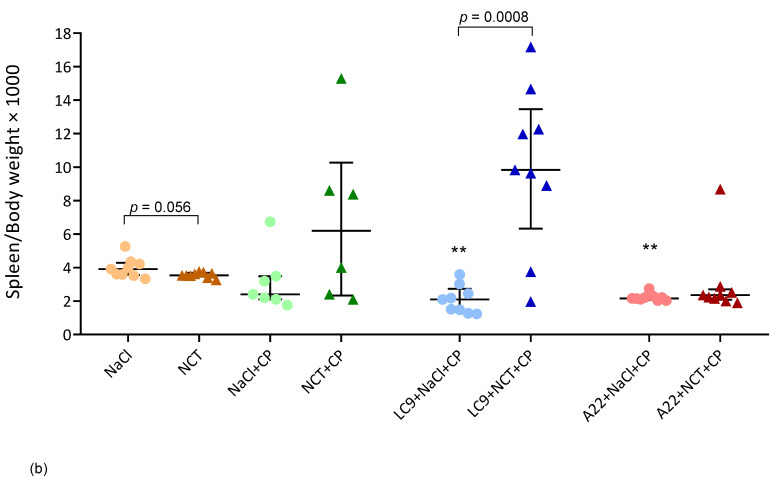
Organ weight per body weight × 1000 at time of death or euthanasia compared to the baseline for the lung (**a**) and the spleen (**b**). Scatter dot plots with the medians and quartiles (*n* = 4–9); ** *p* < 0.01 versus plain NaCl (Kruskal-Wallis test); *p*-values < 0.1 between each test and control group are shown (Mann–Whitney test).

Organ weights were investigated as a measure of the inflammatory reaction (lung weight) and the immune response (spleen weight). Normalized lung and spleen weights (organ weight × 1000/body weight) were different between the NCT and saline groups in the mice infected with *L. corymbifera*. The lungs had a lower weight in the NCT group, with the median values of 8.84 (quartiles, 8.42, 9.71) than in the saline group with 11.42 (quartiles, 10.32, 12.43) (*p* = 0.0005, *n* = 9) (Figure 3a). The spleens, by contrast, had a much higher weight in the NCT group with a median of 9.83 (quartiles, 6.33, 13.47) than in the saline group with a median of 2.10 (quartiles, 1.38, 2.74) (*p* = 0.0008, *n* = 9) (Figure 3b). Remarkably, the animals treated with only cyclophosphamide and inhalations but no fungi also showed a lower lung weight in the NCT group with a median of 8.05 (quartiles, 7.5, 8.8) than in the saline one with a median of 9.03 (quartiles, 8.82, 9.80) (*p* = 0.035) (Figure 3a). In the mice infected with *A. fumigatus*, no differences were seen between the test and control group.

### 3.6. Body Weight

**Figure 4:**
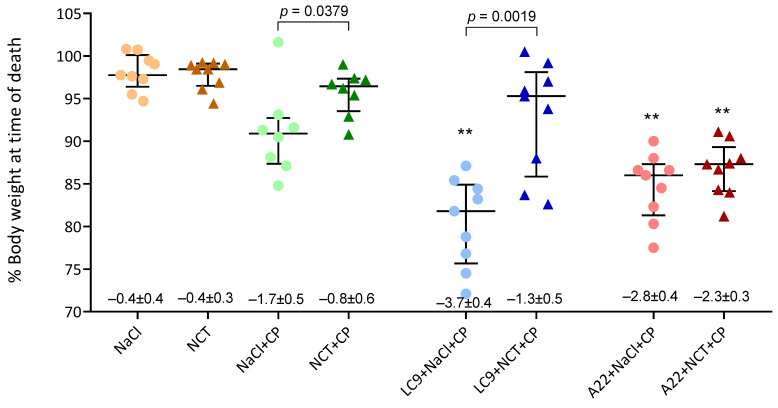
Percentage of body weight at the time of death or euthanasia compared to the baseline. Scatter dot plots with the medians and quartiles (*n* = 8–9). The absolute weight loss in grams as the mean values and SD is indicated below the panels; ** *p* < 0.01 versus plain NaCl (Kruskal–Wallis test); *p*-values < 0.1 between each test and control group are shown (Mann–Whitney test). Of note, the two mice with the lowest weight in group 6 (*L. corymbifera*, NCT) were the only ones who died in this group before the endpoint of 15 days.

Body weight and body temperature were measured as parameters for the intensity of inflammation in the body of mice. The animals of all the groups lost body weight at the time of their exitus compared to time zero (Figure 4). The lowest absolute loss occurred in the animals treated only with inhalations of saline (*p* = 0.029) and NCT (*p* = 0.006) with 0.4 g each and no difference between these groups (*p* = 0.78). The loss of weight in all the other groups was higher.

The percentage of body weight at the time of death of the animals compared to the baseline is shown in Figure 4. It was markedly higher in the NCT group than in the control group in the animals infected with *L. corymbifera* (*p* = 0.0019). Of note, a similar difference was seen in non-infected mice treated with cyclophosphamide and inhalations (*p* = 0.0379). No difference was found between the *A. fumigatus* groups.

### 3.7. Body Temperature

The body temperature markedly dropped by 3.2–9.1 °C in the mice infected with *A. fumigatus* before their death in both the test and control groups. Only the one surviving mouse in the NCT group had stable temperature over 15 days. In the mice infected with *L. corymbifera*, by contrast, the loss of temperature ranged between 1.3 °C and 3.8 °C in the control group (except for 6.9 °C in one mouse), while it was 0.3–2.7 °C in the test group (*p* = 0.051 between the groups). Of note, cyclophosphamide itself caused an increased rate of temperature loss in the animals not challenged with fungi, which was significantly attenuated by NCT (Figure S1).

### 3.8. Blood Parameterss

Laboratory analyses of the blood taken two days after infection of the mice clearly showed signs of inflammation compared to the immunosuppressed controls receiving no fungi. The median haptoglobin values (quartiles) in the control groups were 0.93 (0.64, 1.13) µg/mL for saline and 1.30 (0.93, 2.13) µg/mL for NCT. In the animals receiving *L. corymbifera*, the values were 2.70 (2.22, 3.07) × 10^3^ µg/mL for saline and 1.83 (0.92, 2.90) × 10^3^ µg/mL for NCT. In the animals receiving *A. fumigatus*, the values were 1.75 (0.84, 2.73) × 10^3^ µg/mL for saline and 1.24 (0.61, 2.21) × 10^3^ µg/mL for NCT. There was no difference between NCT and the respective saline controls and between *Lichtheimia* and *Aspergillus* (*p* > 0.99 each), but between the animals receiving fungi or no fungi (*p* < 0.01). In the surviving animals receiving *Lichtheimia*, the haptoglobin values decreased approximately tenfold on day 8, while they normalized in the NCT group on day 14 (*p* = 0.19 compared to the NCT and saline groups that received no fungus).

**Figure 5:**
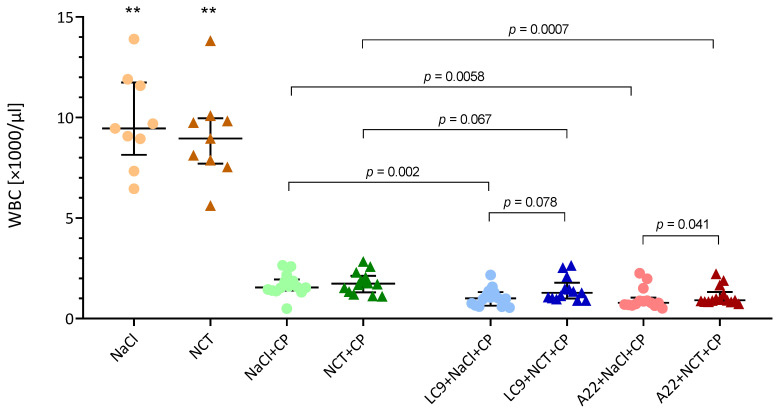
White blood cell count on day 2. Scatter dot plots with the medians and quartiles (*n* = 9–14). Both the plain NCT group and the plain NaCl groups were highly significantly different from all the other groups (** *p* < 0.01, Kruskal–Wallis test). Further *p*-values are indicated (Mann–Whitney test).

Immune suppression highly significantly reduced the white blood cell count (WBC, Figure 5) as well as single counts for granulocytes, monocytes, and lymphocytes (Figure S2) on day 2 (*p* < 0.01, *n* = 9–14). The WBC numbers were even a little lower in the mice treated with *Aspergillus* than in those only immunosuppressed (*p* < 0.01, Figure 5). For *Lichtheimia*, this was only significant in the saline group. Of note, the WBC count was a little higher in the NCT than in the NaCl group in the mice receiving *Lichtheimia* (*p* < 0.1) and *Aspergillus* (*p* < 0.05) (Figure 5). This became even clearer on day 8, when the WBC in the mice receiving only cyclophosphamide and those challenged with *Lichtheimia* was significantly higher in the NCT than in the saline group (*p* = 0.0146 and *p* < 0.01, respectively; *n* = 6–9). No differences in the WBC and single-cell types were found between the mice treated with NCT and saline without immune suppression (*p* > 0.25, *n* = 9), indicating the absence of an effect in healthy animals. 

### 3.9. Bronchoalveolar Lavage

**Figure 6:**
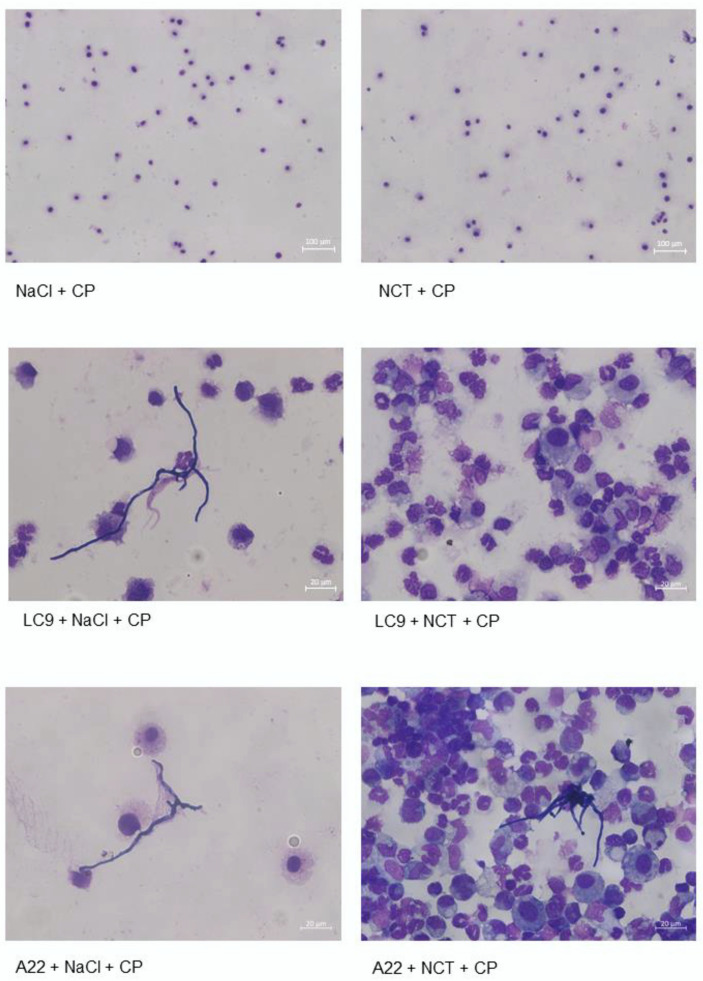
Bronchoalveolar lavage. Samples of BAL of the indicated groups. Normal alveolar macrophages in the immunosuppressed mice that inhaled NaCl or NCT. Inflammation and scattered hyphae in samples from the mice inoculated with molds in addition. Magnification: 100× in the upper panels, 400× in the middle and lower panels.

BAL in the immunocompetent and non-infected mice (groups 1 and 2) showed normal alveolar macrophages with sporadic epithelia without a difference between the NCT and control groups in cell count and morphology on days 2 and 14. The outcome was similar in the immunosuppressed non-infected mice (Figure 6). By strong contrast, in the infected mice on day 2, infiltration by many granulocytes was seen, indicating a strong inflammation. Moreover, scattered short hyphae could be found. These findings were similar for both types of fungi and for both the test and control groups, without significant differences.

### 3.10. Histology 

**Figure 7:**
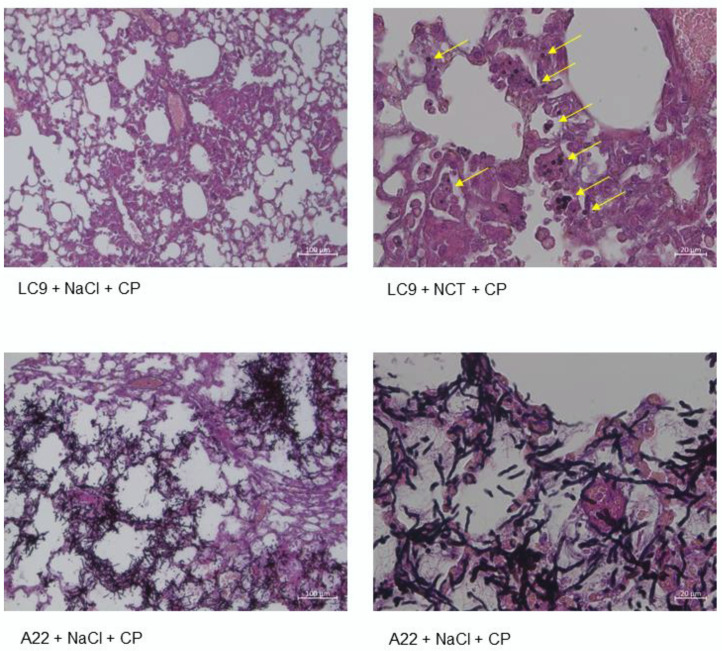
Histology. Acute inflammation in lung samples of the mice infected with *L. corymbifera* (upper panels) and *A. fumigatus* (lower panels) on day 3. Conidia of *Lichtheimia* (arrows) and massive germination of *Aspergillus*. Magnification: 100× in the left panels, 400× in the right ones.

Histological evaluation on day 3 revealed inflammation with granulocyte infiltration in lungs of the mice treated with both species of fungi (Figure 7). Germination and infiltration were massive with *A. fumigatus*, while spores were seen with *Lichtheimia*. No differences were found between the test and control groups on day 3. No pathological findings were found in other organs, i.e., the brain, the kidneys, and the spleen.

Histology of lungs was normal, without pathological findings, on day 14 in all the groups receiving no fungi with or without immune suppression. There were no differences between NCT and saline. 

Histology of reproductive organs (uterus, uterine tube, ovary) performed in the mice that inhaled NCT or saline without further treatment (groups 1 and 2, *n* = 9) did not show any pathological changes or differences between the test and control animals. Only normal variations according to the hormonal cycle were seen.

## 4. Discussion

As a mild active chlorine compound with endogenous origin, NCT proved to be well-tolerated in sensitive body regions, including the lower respiratory system that can be reached via inhalation [14,15,17]. The next step was the evaluation of efficacy of NCT as an anti-infective in lower airway infections. This was not practicable in the pig model, in which the tolerability of inhaled NCT in a lung contaminated with streptococci was demonstrated [16]. Therefore, we chose a fungal pneumonia mouse model already in use at our institute [18], which we combined with equipment for mouse inhalation similar to that used in our previous tolerability study [15]. 

The concentration of 1% NCT was chosen since it turned out to be well-tolerated and effective in previous preclinical and clinical studies [10,11,17]. The rationale for the dosing of three times per day for 10 min is the short period of activity of NCT which immediately drops after the end of inhalation and provides a chlorine cover on the mucosa for additional 10 min [14,15,17]. Moreover, it should provide a convenient dosing schedule for humans. It turned out to be well-tolerated and effective. Significantly lower concentrations of NCT such as 0.1% are inactivated at a relatively high rate in the presence of organic load such as in body fluids or exudates by reaction with reducing substances [25,26]. 

A critical parameter for the success of the mouse pneumonia model is the appropriate fungal load applied to the nostril. If it is too high, as in the present study with *A. fumigatus*, the infection is overwhelming, and all animals die within a short time; here, within 1–4 days. This probably has to do with hyperinflammation in the lung refractory to treatment since no difference between NCT and saline could be found in all the parameters. Since *A. fumigatus* does not show a lower killing rate by NCT than other molds [25,27], we do not think that it was a problem of too little susceptibility. Accordingly, the strain used in the present study (A22) demonstrated a killing curve typical for aspergilli in the presence of NCT in vitro.

With *L. corymbifera*, by strong contrast, where the inoculum was suitable to induce a prolonged infection, the therapy with NCT was successful. Notably, the SUP agar used for *L. corymbifera* allows growth of bacteria, too, which disclosed a bacterial coinfection in most of the mice, presumably stimulated by the immunosuppression. In contrast to the controls, the numbers of bacteria found in the lungs and other organs came to zero in the NCT group in the animals not euthanized on schedule after two days. This clearly demonstrates high efficacy of NCT in the reduction of bacteria in addition to the fungi.

All the main parameters, survival, load of viable pathogens in the lungs and other organs, body weight, organ weight, body temperature, and blood parameters (WBC, haptoglobin) were improved in the NCT group compared to the placebo group, largely with high significance. Low body weight, inappetence, and low temperature are indicators of increased levels of proinflammatory cytokines, which will be antagonized by the antifungal effects of NCT and possibly also by its anti-inflammatory ones (see below). The lower organ weight of the lungs of mice in the test group may be explained by less infiltration and exudate, although this could not clearly be seen in histological and BAL samples compared to the control group. The number of the latter samples, however, was limited since we used the available material more for microbiological cultures, which were estimated to be more important. The higher weight of the spleen in the NCT group could originate from a better immune reaction despite the influence of cyclophosphamide, although the mechanism remains unclear presently.

The rationale for an early start of treatment (1 h after inoculation) was to reach at least a part of the fungal spores by inhalation and influence the infection before occurrence of a marked damage of tissue. If this assumption is decisive remains unclear. Notably, in the three animals euthanized according to the schedule on day two and subjected to quantitative cultures from organ homogenates, two of them in both the NCT and saline groups had high numbers of *Lichtheimia* (4.5–4.8 log_10_/mL) and all three high numbers of bacteria (2.9–5.6 log_10_/mL) in their lungs with no difference between the groups. This indicates the development of a full pneumonia in the test group, too, which underlines the expressiveness of the model.

The number of pathogens (fungi and bacteria) in the lungs correlated well with the outcome in single mice observed for 15 days, which was obvious in the control group. From the two mice with the lowest numbers of *L. corymbifera* (2.34 log_10_/mL each) and no detectable bacteria, one survived and the other one died late on day 13. All the other control mice had high numbers of fungi and/or bacteria and died earlier. In the test group, the correlation was not so clear. Although the mouse with the highest number (2.20 log_10_/mL) died on day 11, no fungi grew in the second one that died on day 10. Naturally, the variability of low numbers near the detection limit is high, which renders correlations with survival difficult. 

The methodology of organ homogenization with Lysing Matrix M 2 mL tubes in a FastPrep-24 5G homogenizer (MP Biomedicals Germany GmbH, Eschwege, Germany) proved to be easy and rapid and did not decrease the viability of fungi and bacteria. It allows a much higher sample throughput than using a stirrer for homogenization and needs much less effort. For plating with a spiral plater, a careful 1:1 dilution of the homogenate in saline is of great advantage to avoid clumping in the small tubing system of the apparatus. This is particularly true for the lungs, the kidneys, and the spleen. The mentioned M2 mL tubes with one large ball inside and shaking for 20 s proved to be suited for our tests. They can be adjusted, however, for different applications since a panel of balls is available and intensity and duration of shaking are variable. 

Molds require 4–8 h of killing time by 1% NCT compared to 10–20 min for bacteria at 37 °C in a buffer solution [27,28]. Their higher resistance may explain the fact that in the majority of surviving mice, low numbers of fungi were still found in the NCT group, while the number of bacteria was below the detection limit. What renders the marked therapeutic effect of NCT even more plausible is its unique marked enhancement of microbicidal activity by the organic material present in body fluids and exudates. This is based on chlorine transfer from NCT to amino groups present in these fluids and formation of the corresponding chloramines in equilibrium (transhalogenation, transchlorination [1,29]). Particularly, formation of monochloramine (NH_2_Cl), which penetrates microorganisms better than NCT because of its higher lipophilicity, plays a role, above all against fungi [25,27,28,30]. In addition, a postantifungal effect (lag of regrowth) after contact with NCT for a sublethal time caused by a chlorine cover may play a role in attenuation of the infection [28,31]. 

As expected from previous tolerability studies in healthy mice and piglets [14,15], piglets with inflamed lungs [16], and healthy humans [17], tolerability of the inhalations with 1% NCT was very good, without signs of discomfort or histological changes in organs or suspicious changes of blood parameters. This was true for both the lungs, which directly came into contact with NCT, and the other organs, to which a transfer of NCT via the bloodstream can be excluded. Small amounts that theoretically might come into contact with the bloodstream would be immediately inactivated by reducing compounds in the blood [32]. 

For regulatory reasons in the drug development, the reproductive organs were investigated histologically with the expected absence of differences from the controls. At most, small amounts of taurine may be absorbed into the blood stream, which are below the detection limit [14,16,17]. On the contrary, taurine can be applied systemically in high doses without toxicity [33,34], and it protects from reproduction toxicity induced by different test substances (e.g., [35,36,37]). This was also true for orally administered NCT in male rats challenged with azathioprine [38], which must be assumed to exert this effect via its decay product taurine. The present study supplements the absence of toxic effects to the reproductive organs in female mammals if NCT is topically applied to another body region. The easy explanation is its reduction at the site of application so that only systemic uptake of taurine is conceivable, but this vanishes in its natural level [14,17]. If small amounts of NCT were taken up to the bloodstream, they would be immediately reduced by antioxidants in the blood forming taurine, too [14,17]. 

An interesting result was the partial reversal of cyclophosphamide-induced negative effects by NCT, such as reversal of weight loss, of the drop of body temperature, of the increased lung weight and low WBC. It can be assumed that this is caused by the well-known anti-inflammatory effects of NCT. These are mainly inactivation of nuclear factor kappa B (NFkappaB), downregulation of tumor necrosis factor alpha, prostaglandin E2, some interleukins (1 beta, 2, 6, 8, 12), collagenase, neopterin, myeloperoxidase, macrophage inflammatory protein-2, upregulation of hemoxygenase-1 and of anti-inflammatory macrophages [5,8,39,40,41]. Actually, significant clinical results with anti-inflammatory effects of NCT were seen in animal studies in vivo. In an earlier mouse study, effects of NCT on the onset and incidence of collagen-induced arthritis were found [42]. More recently, oral administration of NCT protected against experimental mouse colitis and was accompanied with upregulation of nuclear factor erythroid 2-related factor 2 (Nrf2)-dependent cytoprotective and anti-inflammatory gene expression such as heme oxygenase-1 [6]. Additionally, downregulation of NFkappaB and STAT3 and, therefore, proinflammatory factors occurred [6]. By similar mechanisms, topically applied NCT inhibited skin photodamage, i.e., oxidative damage and apoptotic cell death caused by ultraviolet B light in mice [7]. Most recently, it was found that intraperitoneally injected NCT reduced inflammation in LPS-induced pneumonia in high-fat diet-induced obese mice [43]. In the present study, the topical effect in the lungs caused by NCT obviously was sufficient to partly reverse cyclophosphamide damage. In addition, the reaction product of NCT, taurine, which can be absorbed and distributed systemically, might contribute due to its antioxidative and anti-inflammatory properties [8]. It is, however, unclear if taurine reached a sufficient elevation of levels in our model since measurements were not possible because of limitations of sample volumes. In another study, this seems plausible. Pretreatment with oral NCT (100 mg/kg body weight for 6 weeks) significantly abrogated toxic effects of the following treatment with oral azathioprine (5 mg/kg for 4 weeks) to the testes and sperm of albino rats [38]. Topically applied NCT, however, underlies topical decay and traces coming into contact with the bloodstream are reduced immediately. Only high (millimolar) concentrations of NCT mixed directly with blood may overcome the antioxidative capacity in these surroundings and maintain, for instance, antimicrobial activity [32]. Therefore, the systemic effects observed after a local application of NCT must be regarded to be due to taurine and possibly further reaction products. 

Summing up, this study for the first time proved a marked therapeutic effect of inhaled NCT in pneumonia caused by pathogens in vivo. In the fungal mouse model, it is crucial to meet an infection dose that leads to a delayed mortality over two weeks and not to an immediate maximum spore load that kills the animals rapidly within 1–3 days. We were successful with *L. corymbifera*. NCT reduced mortality within the observation period of 15 days, as well as the fungal and concomitant bacterial loads, and it improved all the other major parameters. The application was safe and well-tolerated, with the absence of topical or systemic toxicity including histology of the reproductive organs. Further development of NCT as an endogenous broad-spectrum antiseptic and anti-infective for the lower and upper airways as well is strongly indicated.

## Figures and Tables

**Table 1:** Test and control groups.

Group No.	Infection with fungi	Cyclophosphamide	Treatment	Number of Mice
1	No	No	NaCl	9
2	No	No	NCT	9
3	No	Yes	NaCl	14
4	No	Yes	NCT	14
5	*L. corymbifera*	Yes	NaCl	14
6	*L. corymbifera*	Yes	NCT	14
7	*A. fumigatus*	Yes	NaCl	14
8	*A. fumigatus*	Yes	NCT	14

## Data Availability

Not applicable.

## Supplementary Materials

The following supporting information can be downloaded at: https://www.mdpi.com/article/10.3390/jof8050535/s1, Figure S1: Reduction of body temperature per day in groups receiving no fungi. Figure S2: Single blood cell counts on day 2.
