# Supplementary material for: Efficacy of Inhaled N-Chlorotaurine in a Mouse Model of Lichtheimia corymbifera and Aspergillus fumigatus Pneumonia
Source: J Fungi (Basel). 2022 May 20;8(5):535. doi: 10.3390/jof8050535 (PMC9143854; doi:10.3390/jof8050535)
Supplement: Supplementary file 1 [file jof-08-00535-s001.zip › jof-1725406-supplementary.pdf]

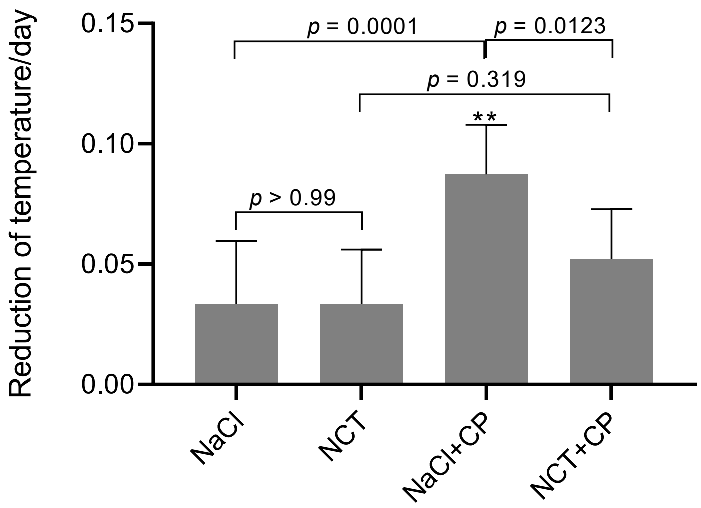

Figure S1. Reduction of body temperature per day in groups receiving no fungi. The average daily drop of body temperature for test and control groups treated with NCT or saline with or without cyclophosphamide is shown. Mean values ± SD of $n = 9$. Student's unpaired *t* test was used for comparison between single groups. ** $p < 0.01$ versus all other groups by one-way ANOVA and Dunnett's multiple comparison test.

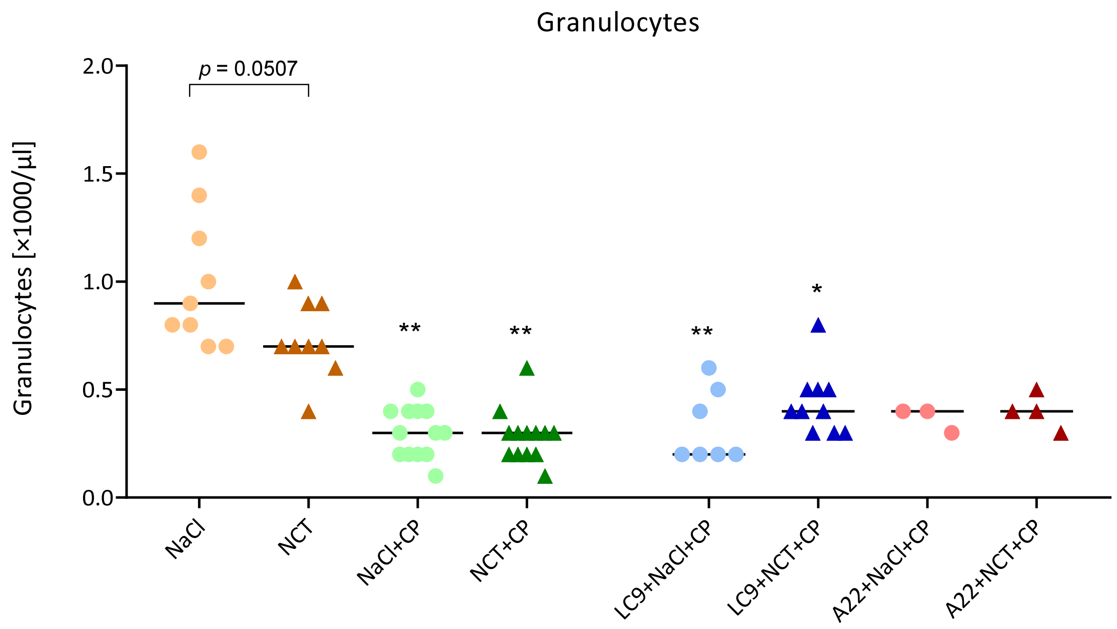

Granulocytes
Granulocytes [×1000/µl]
2.0
1.5
1.0
0.5
0.0
p = 0.0507
**
**
**
*
NaCl
NCT
NaCl+CP
NCT+CP
LC9+NaCl+CP
LC9+NCT+CP
A22+NaCl+CP
A22+NCT+CP

(a)

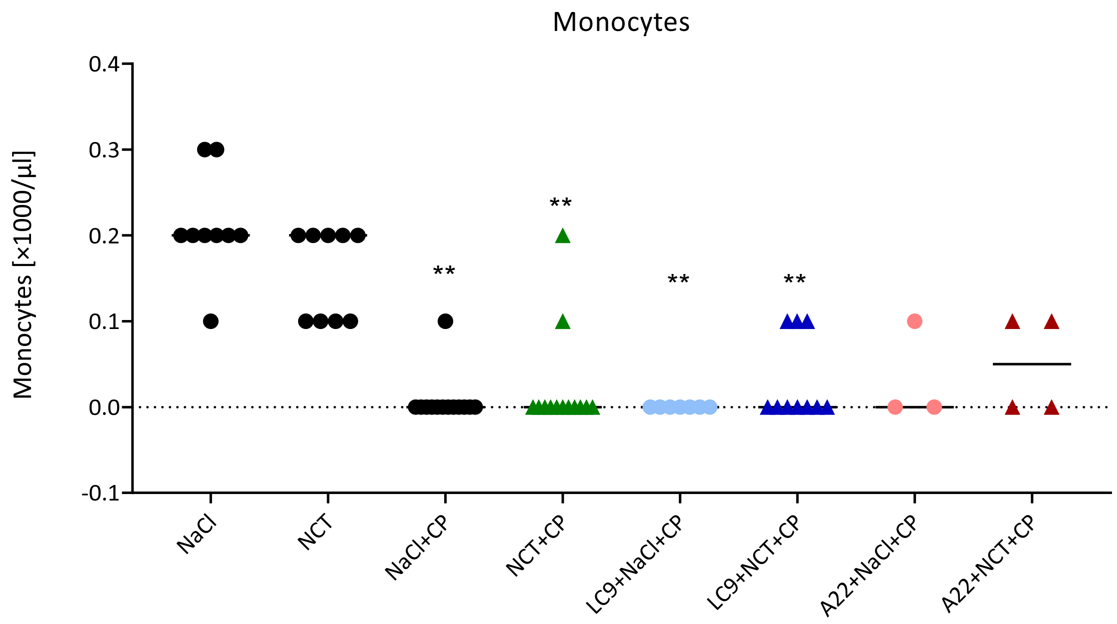

Monocytes
Monocytes [×1000/µl]
0.4
0.3
0.2
0.1
0.0
-0.1
**
**
**
**
NaCl
NCT
NaCl+CP
NCT+CP
LC9+NaCl+CP
LC9+NCT+CP
A22+NaCl+CP
A22+NCT+CP

(b)

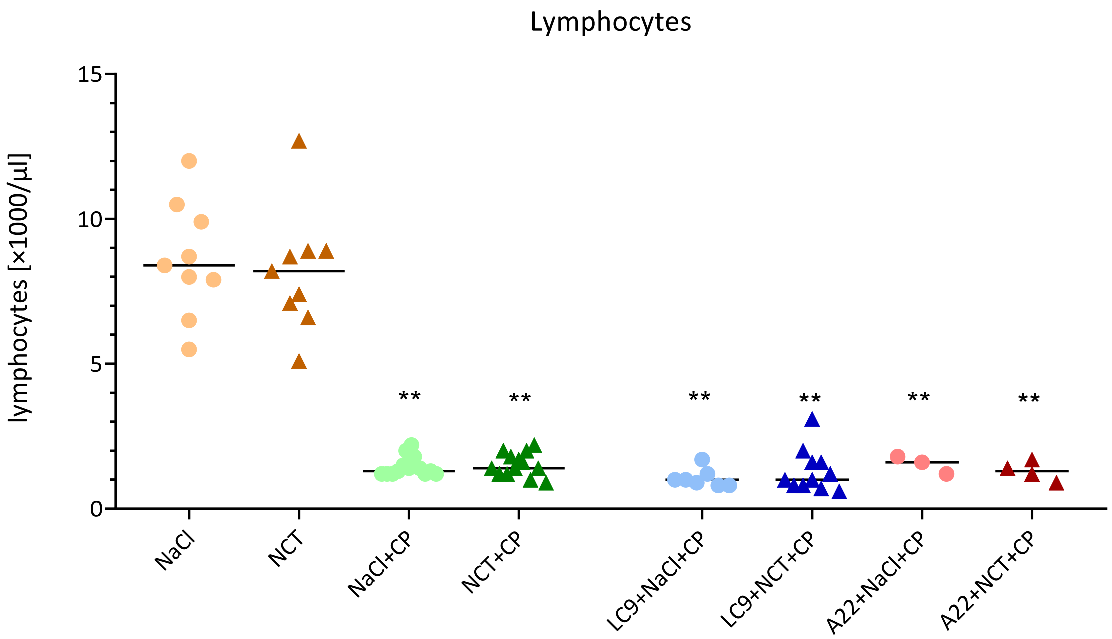

(c)

Figure S2. Single blood cell counts on day 2. Scatter dot plots with medians and quartiles (*n* = 3-13) for granulocytes (**a**), monocytes (**b**) and lymphocytes (**c**). * $p < 0.05$ and ** $p < 0.01$ versus plain NaCl (Kruskal-Wallis test); p values < 0.1 between each test and control group are shown (Mann-Whitney test).
